# Supplementary material for: Estimation of mean population salt intakes using spot urine samples and associations with body mass index, hypertension, raised blood sugar and hypercholesterolemia: Findings from STEPS Survey 2019, Nepal
Source: PLoS One. 2022 Apr 12;17(4):e0266662. doi: 10.1371/journal.pone.0266662 (PMC9004746; doi:10.1371/journal.pone.0266662)
Supplement: Attachment — Submitted filename: PONE-D-21-06676_reviewer.pdf [file pone.0266662.s003.pdf]

PLOS ONE

Estimation of mean Population salt intakes using Spot Urine samples and its association to BMI, Hypertension, raised Blood Sugar and Blood Lipids: Findings from Non-communicable Disease Risk Factors (STEPS) Survey 2019 in Nepal

--Manuscript Draft--

**Manuscript Number:**	PONE-D-21-06676
**Article Type:**	Research Article
**Full Title:**	Estimation of mean Population salt intakes using Spot Urine samples and its association to BMI, Hypertension, raised Blood Sugar and Blood Lipids: Findings from Non-communicable Disease Risk Factors (STEPS) Survey 2019 in Nepal
**Short Title:**	Associations of urinary sodium levels with BMI, Hypertension, raised Blood Sugar and Blood Lipids in Nepal
**Corresponding Author:**	Meghnath Dhimal, Ph.D.<br>Nepal Health Research Council<br>Kathmandu, Bagmati NEPAL
**Keywords:**	Spot Urine, Salt Intake, INTERSALT, BMI, Hypertension, raised Blood Sugar and Blood Lipids, Nepal
**Abstract:**	Abstract<br>Background: High dietary salt intake is recognized as a risk factor for several noncommunicable diseases (NCDs), in particular cardiovascular diseases (CVDs), including heart attack and stroke. Accurate measurement of salt intake is essential for setting realistic goals and plans for salt reduction strategies. We used spot urine sample for the first time to estimate the mean population salt intake in Nepal. We also evaluate the association of salt intake with BMI, Hypertension, raised Blood Sugar and Blood Lipids and their relation with socio-demographic characteristics.<br>Methods: A population based cross sectional study was carried out from February to May 2019 using a WHO STEP-wise approach to surveillance. 4361 (67.6%) spot urine was collected from the men and women aged 15–69 for the analysis of salt intake. INTERSALT equation was used to calculate population salt intake. Student's‘t’ test, ANOVA and multivariate linear regression regressions was used to assess the association between salt intake with explanatory factors. Statistical significance was accepted at P <.05.<br>Results: The average (±SD) age of participants was 40 (14.1) years. Mean salt intake was estimated to be 9.1g/d derived from spot urine samples. A total of 69.4% of the population consumed more than the WHO’s recommended amount of 5 g salt per day, with almost half of the population 48.9% consuming more than 10 g of salt per day. Higher salt intake was significantly associated with male gender (β for male = 0.98g; 95% CI: 0.87, 1.1) and younger age groups (β 25-39 years = 0.08; 95% CI: -0.08, 0.23) and higher BMI (β = 0.19; 95% CI: 0.18, 0.21). Participants who were hypertensive, and had high cholesterol ate less salt than people who had normal blood pressure and cholesterol level (P==0.000).<br>Conclusions: These findings make a strong case for action to reduce salt consumption in Nepal to achieve the global target of a 30% reduction in population salt intake by 2025. Our study clearly highlighted the need of future studies using longitudinal data or randomized clinical trials to assess the role of dietary salt in the development / prevention of blood pressure, diabetes mellitus and cholesterol levels in Nepalese population.
**Order of Authors:**	Saroj Bhattarai
	Bihungum Bista
	Binod Kumar Yadav
	Pradip Gyanwali
	Anil Poudyal
	Anjani Kumar Jha

Powered by Editorial Manager® and ProduXion Manager® from Aries Systems Corporation

	Meghnath Dhimal
**Additional Information:**	
**Question**	**Response**
**Financial Disclosure**<br><br>Enter a financial disclosure statement that describes the sources of funding for the work included in this submission. Review the submission guidelines for detailed requirements. View published research articles from *PLOS ONE* for specific examples.<br><br>This statement is required for submission and **will appear in the published article** if the submission is accepted. Please make sure it is accurate.<br><br>**Unfunded studies**<br>Enter: *The author(s) received no specific funding for this work.*<br><br>**Funded studies**<br>Enter a statement with the following details:<br>• Initials of the authors who received each award<br>• Grant numbers awarded to each author<br>• The full name of each funder<br>• URL of each funder website<br>• Did the sponsors or funders play any role in the study design, data collection and analysis, decision to publish, or preparation of the manuscript?<br>• **NO** - Include this sentence at the end of your statement: *The funders had no role in study design, data collection and analysis, decision to publish, or preparation of the manuscript.*<br>• **YES** - Specify the role(s) played.<br><br>* typeset	The author(s) received no specific funding for this work.
**Competing Interests**<br><br>Use the instructions below to enter a competing interest statement for this submission. On behalf of all authors, disclose any competing interests that could be perceived to bias this work—acknowledging all financial support and any other relevant financial or non-financial competing interests.	The authors have declared that no completing interests exist.

Powered by Editorial Manager® and ProduXion Manager® from Aries Systems Corporation

This statement is required for submission and **will appear in the published article** if the submission is accepted. Please make sure it is accurate and that any funding sources listed in your Funding Information later in the submission form are also declared in your Financial Disclosure statement.<br><br>View published research articles from *PLOS ONE* for specific examples.<br><br>**NO authors have competing interests**<br><br>Enter: *The authors have declared that no competing interests exist.*<br><br>**Authors with competing interests**<br><br>Enter competing interest details beginning with this statement:<br><br>*I have read the journal's policy and the authors of this manuscript have the following competing interests: [insert competing interests here]*<br><br>* typeset	
**Ethics Statement**<br><br>Enter an ethics statement for this submission. This statement is required if the study involved:<br><br>• Human participants<br>• Human specimens or tissue<br>• Vertebrate animals or cephalopods<br>• Vertebrate embryos or tissues<br>• Field research<br><br>Write "N/A" if the submission does not require an ethics statement.<br><br>General guidance is provided below. Consult the submission guidelines for detailed instructions. **Make sure that all**	The study was approved by the Ethical Review Board (ERB) of Nepal Health Research Council, Katmandu, Nepal.

Powered by Editorial Manager® and ProduXion Manager® from Aries Systems Corporation

**information entered here is included in the Methods section of the manuscript.**<br><br>**Format for specific study types**<br><br>**Human Subject Research (involving human participants and/or tissue)**<br>• Give the name of the institutional review board or ethics committee that approved the study<br>• Include the approval number and/or a statement indicating approval of this research<br>• Indicate the form of consent obtained (written/oral) or the reason that consent was not obtained (e.g. the data were analyzed anonymously)<br><br>**Animal Research (involving vertebrate animals, embryos or tissues)**<br>• Provide the name of the Institutional Animal Care and Use Committee (IACUC) or other relevant ethics board that reviewed the study protocol, and indicate whether they approved this research or granted a formal waiver of ethical approval<br>• Include an approval number if one was obtained<br>• If the study involved *non-human primates*, add *additional details* about animal welfare and steps taken to ameliorate suffering<br>• If anesthesia, euthanasia, or any kind of animal sacrifice is part of the study, include briefly which substances and/or methods were applied<br><br>**Field Research**<br><br>Include the following details if this study involves the collection of plant, animal, or other materials from a natural setting:<br>• Field permit number<br>• Name of the institution or relevant body that granted permission	
**Data Availability**<br><br>Authors are required to make all data underlying the findings described fully available, without restriction, and from the time of publication. PLOS allows rare exceptions to address legal and ethical	Yes - all data are fully available without restriction

Powered by Editorial Manager® and ProduXion Manager® from Aries Systems Corporation

concerns. See the PLOS Data Policy and FAQ for detailed information.<br><br>A Data Availability Statement describing where the data can be found is required at submission. Your answers to this question constitute the Data Availability Statement and **will be published in the article**, if accepted.<br><br>**Important:** Stating 'data available on request from the author' is not sufficient. If your data are only available upon request, select 'No' for the first question and explain your exceptional situation in the text box.<br><br>Do the authors confirm that all data underlying the findings described in their manuscript are fully available without restriction?	
**Describe where the data may be found in full sentences. If you are copying our sample text, replace any instances of XXX with the appropriate details.**<br><br>• If the data are **held or will be held in a public repository**, include URLs, accession numbers or DOIs. If this information will only be available after acceptance, indicate this by ticking the box below. For example: *All XXX files are available from the XXX database (accession number(s) XXX, XXX.*).<br>• If the data are all contained **within the manuscript and/or Supporting Information files**, enter the following: *All relevant data are within the manuscript and its Supporting Information files.*<br>• If neither of these applies but you are able to provide **details of access elsewhere**, with or without limitations, please do so. For example:<br><br>*Data cannot be shared publicly because of [XXX]. Data are available from the XXX Institutional Data Access / Ethics Committee (contact via XXX) for researchers who meet the criteria for access to confidential data.*	The data for this study was obtained from Non Communicable Diseases Risk Factors: STEPS Survey Nepal 2019. The data and other additional information about this study can be received upon request to Nepal Health Research Council (NHRC) Ramshah Path, Kathmandu, Nepal or corresponding author of this paper on reasonable request (email: Dr. Meghnath Dhimal, meghdhimal@nhrc.gov.np)

Powered by Editorial Manager® and ProduXion Manager® from Aries Systems Corporation

The data underlying the results presented in the study are available from (include the name of the third party and contact information or URL).<br>• This text is appropriate if the data are owned by a third party and authors do not have permission to share the data.<br><br>* typeset	
Additional data availability information:	

Powered by Editorial Manager® and ProduXion Manager® from Aries Systems Corporation

Manuscript

Click here to access/download;Manuscript;Original Article_Salt.docx

Original Article

Estimation of mean Population salt intakes using Spot Urine samples and its association to BMI, Hypertension, raised Blood Sugar and Blood Lipids: Findings from Non-communicable Disease Risk Factors (STEPS) Survey 2019 in Nepal

Running title: Associations of urinary sodium levels with BMI, Hypertension, raised Blood Sugar and Blood Lipids

List of authors:

Saroj Bhattarai[1], Bihungum Bista[1], Binod Kumar Yadav[2], Pradip Gynawali[1], Anil Poudyal[1], Anjani Kumar Jha[1], Meghnath Dhimal[1*]

[1]Nepal Health Research Council, Ramshah Path, Kathmandu, Nepal, bhattaraisaroj23@gmail.com

[2]Department of Biochemistry, Maharajgunj Medical Campus, Institute of Medicine, Tribhuvan University, Maharajgunj, Kathmandu, Nepal, binod3aug@gmail.com

***Corresponding Author:**

Dr. Meghnath Dhimal, Chief/Senior Research Officer, Nepal Health Research Council, Goverment of Nepal, Ministry of Health Complex, Ramshah Path, Kathmand,Nepal, Phone No. 0097714254220, Fax No. 0097714262469, Email: meghdhimal@nhrc.gov.np; meghdhimal@gmail.com

Abstract

Background: High dietary salt intake is recognized as a risk factor for several non-communicable diseases (NCDs), in particular cardiovascular diseases (CVDs), including heart attack and stroke. Accurate measurement of salt intake is essential for setting realistic goals and plans for salt reduction strategies. We used spot urine sample for the first time to estimate the mean population salt intake in Nepal. We also evaluate the association of salt intake with BMI, Hypertension, raised Blood Sugar and Blood Lipids and their relation with socio-demographic characteristics.

Methods: A population based cross sectional study was carried out from February to May 2019 using a WHO STEP-wise approach to surveillance. 4361 (67.6%) spot urine was collected from the men and women aged 15–69 for the analysis of salt intake. INTERSALT equation was used to calculate population salt intake. Student's't' test, ANOVA and multivariate linear regression regressions was used to assess the association between salt intake with explanatory factors. Statistical significance was accepted at P<.05.

Results: The average (±SD) age of participants was 40 (14.1) years. Mean salt intake was estimated to be 9.1g/d derived from spot urine samples. A total of 69.4% of the population consumed more than the WHO's recommended amount of 5 g salt per day, with almost half of the population 48.9% consuming more than 10 g of salt per day. Higher salt intake was significantly associated with male gender (β for male = 0.98g; 95% CI: 0.87, 1.1) and younger age groups ($\beta_{25\text{-}39 \text{ years}}$ = 0.08; 95% CI: -0.08, 0.23) and higher BMI (β = 0.19; 95% CI: 0.18, 0.21). Participants who were hypertensive, and had high cholesterol ate less salt than people who had normal blood pressure and cholesterol level (P==0.000).

Conclusions: These findings make a strong case for action to reduce salt consumption in Nepal to achieve the global target of a 30% reduction in population salt intake by 2025. Our study clearly highlighted the need of future studies using longitudinal data or randomized clinical trials to assess the role of dietary salt in the development / prevention of blood pressure, diabetes mellitus and cholesterol levels in Nepalese population.

Keywords: Spot Urine, Salt Intake, INTERSALT, BMI, Hypertension, raised Blood Sugar and Blood Lipids, Nepal

Introduction

Sodium in the salt is the principal cation in extracellular fluid in the body, and is an essential nutrient necessary for normal cell function and for neurotransmission [1,2,3]. ~~Salt is essential nutrient if consumed in limit.~~ High dietary salt intake is associated with high blood pressure which is recognized as a risk factor for non-communicable diseases (NCDs), in particular cardiovascular diseases (CVDs), including heart attack and stroke [4,5,6]. Conclusive scientific evidence also found the association of excessive consumption of sodium with osteoporosis, cataract, kidney stones, and diabetes [4,6]. Globally, populations are consuming excessive amounts of salt, with the worldwide estimated mean salt intake being almost 9-12 g/day [7], in many countries and even higher intakes are found in Asia, which is associated with negative effects on health [8].

In Nepal, noncommunicable diseases (NCDs) are estimated to cause 60% of total deaths, with CVDs contributing to 22% of the deaths [9]. The 2019 WHO STEPwise approach to surveillance of non-communicable disease risk factor (STEPS) survey reported almost 24.5% of the Nepalese

population had raised blood pressure [10]. High blood pressure and CVDs place a large economic burden to the government [8,11,12]. The World Health Organization (WHO) recommends consuming less than 2 grams of sodium or 5 grams of salt per day amongst adults [13]. A 30% relative reduction in mean population intake of salt/sodium by 2025 relative to 2010 levels under WHO global action plan is one of the single most effective public health strategies to reduce the burden of NCDs worldwide [13]. Nepal has also incorporated it as one of the key targets in its 5-year multisectoral action plan for 2014-2020 [14]. To achieve this objectives accurate measurement of salt intake is essential for setting realistic goals and plans for salt reduction strategies [15].

For multiple reasons, dietary assessment survey and nutrition databases methods for estimation of salt intake often underestimates sodium intake [16]. The standard approach to measuring the mean salt intake of a population has been the collection of 24-h urine samples on a subset of individuals [17]. However, this method is troublesome, time consuming, costly to participants due to the complex nature of urine sample collection and may miss the sodium estimation excreted through non-urinary routes [18] and some dietary sodium derives from sources other than salt [19]. There has been a growing interest in finding less costly and burdensome alternatives to 24-h urine collection, such as spot urine samples [20]. Equations that use spot urine samples to estimate population salt intake have been explored as a possible alternative in a number of studies [21,22,23,24,25,26,27,28]. For a country to address an estimate of mean national salt intake and, in December 2013, the WHO incorporated measurement of mean population salt intake as an element of the WHO Stepwise approach to Surveillance (STEPS) protocol [29]. Hence; we used spot urine sample data from a nationally representative population

in Nepal for the first time to estimate the mean population salt intake. We also evaluate the association of salt intake with BMI, Hypertension, raised Blood Sugar and Blood Lipids and their relation with socio-demographic characteristics.

Methods

Study design and Sampling Technique

A population based cross sectional study was carried out from February to May 2019 using a WHO STEP-wise approach to surveillance. Survey population includes men and women aged 15–69 years who have been living at their place of residence for at least six months or visitors who stayed in the households the night before the survey are eligible to interview. National representative sample was selected using multistage cluster sampling. Sample size of 925 survey participants were sampled from each of seven provinces with the total sample size of 6475 participants aged 15 to 69 years. The survey listed all the households of 259 primary sample unit (PSUs) 37 PSUs in each of 7 Provinces, 25 households per PSU were sampled using systematic random sampling. From each of the selected household, one adult member of 15-69 years was sampled randomly for participation in the survey using the android tablet.

Data Collection

The survey was conducted using the standardized WHO NCD STEPS questionnaire version 3.2. 60 Field research assistant with public health, nursing, laboratory, health assistant education backgrounds were mobilized all over the Nepal. The data collection process included three steps. Step 1: This step comprised a Questionnaire to gather demographic and behavioral

characteristics of the study population; face to face interview was carried out to fill the questionnaire. In Step 2: Anthropometric measurement: Blood pressure, height, weight, hip, waist circumference were measured. Weight was measured with a portable digital weighing scale (Seca, Germany). Waist and hip circumference was measured using a constant tension tape (Seca, Germany). Blood pressure was measured by using digital, automated blood pressure monitor (OMRON digital device) with a universal size cuff. Step 3: Biochemical measurements were undertaken to determine the proportion of the study population with diabetes, raised blood glucose and abnormal lipid level. Blood glucose and total cholesterol was measured through dry chemistry using CardioCheck PA Analyser as recommended and supported by WHO. Concentrations of glucose, total cholesterol were measured in capillary whole blood. Fasting samples was taken to measure raised blood glucose. Participants were instructed to fast overnight for 12 hours at the time of household visit for Step 1 and 2.

Estimation of 24-hour salt intake based on sport urine testing

For the estimation of the mean population salt intake, STEPs survey utilized spot urine sample as a proxy to 24-hour urine samples. Spot urine collection was done to identify the level of Sodium (Na), potassium (K) and creatinine.

Process

For urine collection, urine container with QR code was provided to participants to collect spot urine. Participants self-collected the urine samples at home before fasting for blood sample collection the next day during their scheduled appointment for biochemical measurements.

The collected urine sample was stored in dark place in normal room temperature until they were transported to the lab. Laboratory setup was done in every province headquarters and nearly located places. Determination of Na and K in urine is carried with Ion-selective Electrodes (ISE) in an automated Analyzer (Beckman Coulter, CA, USA) and creatinine was estimated in urine by the use of semi-automated biochemistry analyser (Nova Biomedical Cooperation, Waltham, MA, USA). The unit of measurements for Na and K was mmol/L, while creatinine was mg/dl. Participants who were pregnant, fasting before collecting the urine sample, having contaminated urine sample with blood were excluded at the time of analysis. Participants whose height was less than 100 cm or above 270 cm; weight was less than 20kg or above 350 kg were also excluded.

Daily salt consumption estimation

Nepal estimated the 24 hours salt intake for the first time, and it was not included in 2013 survey. Three main studies developed the estimation of 24-h urinary sodium intake from spot urine samples that are: Kawasaki, INTERSALT and Tanaka [21,25,27]. However, limited evidence support the preferential use of one equation over another in a given population/context [22,28]. For this survey, Nepal used the INTERSALT Southern European equation to estimate 24 hour mean salt intake because it was developed using large heterogeneous population sample [21].

INTERSALT

For Southern Europe:

Male:

$$\left(20.861 + 0.45 \times Naspot\left(\frac{mmol}{L}\right)\right) - 3.09 \times Crspot\left(\frac{mmol}{L}\right) + 4.16 \times BMI\left(\frac{kg}{m^2}\right) + 0.22 \times Age(year)$$

Female:

$$\left(21.98 + 0.33 \times Naspot\left(\frac{mmol}{L}\right)\right) - 2.44 \times Crspot\left(\frac{mmol}{L}\right) + 2.42 \times BMI\left(\frac{kg}{m^2}\right) + 2.34 \times Age(year) - 0.03 \times Age^2(year)$$

***Naspot*:** Sodium concentration in spot urine (mmol/L)

***Crspot*:** Creatinine concentration in spot urine (mmol/L)

BMI: Body Mass Index

The equations given above compute 24 hour 'sodium' intake, which is then converted to 'salt' intake by the division of 17.1 (or multiplication of 2.54/1000*23) as a conversion factor to obtain the final estimated 24-hour salt intake in grams.

Data processing and analysis

Descriptive statistics for demographic and behavior were recorded for both the salt and the non-salt participants of the STEPS survey. All the analyses for this study were performed on STATA 13.1 version using survey (svy) set command, defining clusters and sampling weight information. The proportion of the population above the WHO-recommended guideline of 5 g/d of salt and sodium to potassium ratio were measured.

Student's‘t’ test, ANOVA and Chi-square statistics were used to determine the association of mean salt consumption levels with explanatory factors. The association between salt intake and participant characteristics were explored using Multivariate linear regression regressions for age, sex, province, education, income, BMI, and Blood Pressure, Diabetes Mellitus and cholesterol. Statistical significance was accepted at $P<.05$.

Ethical consideration

Written informed consent was obtained from research participants before enrolling in the study after explaining the objective of study, voluntary participation, privacy, confidentiality, and their right to withdraw at any point of study. Ethical approval was obtained from the Ethical Review Board (ERB) of the Nepal Health Research Council (NHRC), Government of Nepal.

Results

Response rate

Amongst the initially planned 6475 sample size, 1 PSU with 25 participants was dropped, leaving 6450 as our total sample size. The number of participants consented and completed the survey for STEP 1 was 5593 (86.7%), Step 2 was 5582 (86.5%), and STEP 3 was 5350(86.5%). Among them only 4361 (67.6%) participants consented provides the spot urine for analysis of salt intake. The demographic characteristics of the participants included in this study are displayed in "Table 1".

Characteristics of the 1998 male and 3595 female participants in the study are reported in "Table 1". The average (±SD) age of participants was 40 (14.1) years. Most of the participants (both male and female) reside in Municipality and had none/primary education. Mean BMI of female is slightly greater than that of male (22.8 kg/m^2 compared to 22.6 kg/m^2). Mean blood pressure (BP) was higher in male than female (127.7/83 mm of Hg compared to 121.3/80.3 mm of Hg). Among the male respondents, more than one fourth (29.8%) of population were assessed as

having hypertension. Similarly, among the female respondents about 19.7% respondents had high blood pressure (i.e., SPB ≥140 mm Hg and/or DBP ≥90 mm Hg). More female participants (11.2 %) reported having previously been diagnosed with hypertension on treatment compared to (7.0%) male participants. Diabetes mellitus (DM) was prevalent among male than female (6.3% compared to 5.3) in contrast female respondents had high cholesterol level than male (13.9% compared to 7.7%). More female participants reported of ever had a heart attack or chest pain from heart disease or stroke (1.4 % compared to 0.8%) in male participants.

Table 1. Study population Characteristics

**Characteristics**	**Male**<br>**n (%)**	**n**	**Female**<br>**n (%)**	**n**
**Age** (mean) (SD) 39.9 (14.1)	42.2 (14.6)	1998	38.6 (13.7)	3595
**Age range**				
15-24	13.8	275	15.8	568
25-39	30.8	615	41.0	1,472
40-54	30.5	609	26.8	965
55-69	25.0	499	16.4	590
**Residence**				
Metropolitan/Sub metropolitan city	13.8	276	11.9	429
Municipality	48.3	964	49.8	1,791
Rural municipality	37.9	758	38.3	1,375
**Province**				

Province 1	14.3	285	14.4	519
Province 2	**17.7**	353	12.5	450
Bagmati Province	15.1	302	12.7	457
Gandaki Province	13.4	267	14.6	526
Lumbini Province	13.4	268	14.7	529
Karnali Province	13.1	261	15.2	547
Sudurpashchim Province	13.1	262	15.8	567
**Education**				
None/less than primary	39.6	792	55.6	2,000
Primary	21.2	424	17.4	627
Secondary	23.3	466	17.3	622
More than secondary	15.8	316	9.6	345
**Wealth**				
Lowest	25.2	504	32.0	1,149
Second	18.3	366	19.4	696
Middle	17.3	345	16.8	604
Fourth	16.9	338	15.0	540
Highest	22.3	445	16.9	606
Height (mean) (SD)	161.1 (7.8)	1997	151.8(6.6)	3522
Weight (mean) (SD)	58.7(11.1)	1997	52.6(10.9)	3522
Waist circumferences (mean) (SD)	80.4 (11)	1997	79.9 (17.9)	3522
BMI (mean) (SD)	22.6 (3.8)	1992	22.8 (4.2)	3507
Normal (BMI 18.5-24.9)	65.9	1992	65.1	3507

Underweight (BMI<=18.4)	10.7	1992	9.8	3507
Overweight (BMI 25.0-29.9)	20.2	1992	19.8	3507
Obese (BMI >= 30.0)	3.2	1992	5.3	3507
SBP (mean) (SD)	127.7 (17.9)	1997	121.3 (17.3)	3585
DBP (mean) (SD)	83.0(11.6)	1997	80.3(10.5)	3585
BP (High)	29.8	1966	19.7	3540
People measured to be hypertensive on treatment	7.9	721	11.2	817
Diabetes Mellitus	6.3	1834	5.3	3357
Cholesterols (High)	7.7	1904	13.9	3438
Ever had a heart attack or chest pain from heart disease or stroke	0.8	1998	1.4	3595

SD, Standard deviation

Mean salt intake was estimated to be 9.1g/d derived from spot urine samples "Table 2". Males had significantly higher salt intake than females (9.6 g/d compared to 8.7 g/d). A total of 69.4% of the population consumed more than the WHO's recommended amount of 5 g salt per day, with almost half of the population 48.9% consuming more than 10 g of salt per day. The sodium/potassium ratio for the population was 3.4 (SE, 0.2).

Table 2. Weighted Results for Salt Intake, Potassium Intake, and Sodium/Potassium Ratio

Salt intake, mean (S.E), g	**Overall (4361)**	**Male (1600)**	**Female (2761)**

	9.1(0.1)	9.6(0.1)	8.7(0.1)
Salt intake above the 5 g WHO target, %	69.4	41.0	59.0
Salt intake above the 10 g WHO target, %	31	64.3	35.7
Potassium intake, mean (S.E), mmol	48.9(0.2)	52.1(1.8)	46.0(1.5)
Sodium intake, mean (S.E), mmol	117.8(3.0)	113.3(3.3)	121.8(3.3)
Sodium/potassium ratio, mean (S.E)	3.7	3.4(0.2)	4.1(0.2)
Creatinine, mean (S.E), mmol	3.4(0.2)	3.3(0.2)	3.4(0.2)

S.E, Standard error

"Table 3" shows analysis on level of salt intake in relation to Participants' Characteristics. Salt intake was found to differ significantly between sex, age range, education, province, BMI, and cholesterol level of participants, which is significant at the 5% level of significance. However; there was no evidence of an association of salt consumption with hypertension, diabetes mellitus and wealth index of participants.

Table 3. Association between Levels of Salt Intake with Participants' Characteristics

**Group**	**Overall (n)**	**Mean, 95%-CI**	**P value**
**Sex**			
Women	2761	8.8 (8.7-8.9)	0.000
Men	1600	9.6 (9.5-9.7)	
**Age range**			

15-24	614	9.0 (8.8-9.1)	0.000
25-39	1617	9.3 (9.2-9.4)	
40-54	1245	9.2 (9.1-9.3)	
55-69	885	8.7 (8.5-8.8)	
**Education**			
None/less than primary	2152	8.9 (8.9-9.0)	0.000
Primary	829	9.2 (9.1-9.4)	
Secondary	858	9.3 (9.2-9.4)	
More than secondary	522	9.2 (9.1-9.4)	
**Wealth index**			
Lowest	1281	9.1 (9.0-9.2)	0.257
Second	831	9.2 (9.1-9.3)	
Middle	749	9.0 (8.9-9.2)	
Fourth	670	9.0 (8.9-9.2)	
Highest	830	9.1 (9.0-9.2)	
**Province**			
Province 1	711	9.1 (8.9-9.2)	0.000
Province 2	713	8.9 (8.8-9.0)	
Bagmati Province	674	9.0 (8.9-9.1)	
Gandaki Province	726	9.1 (8.9-9.2)	
Lumbini Province	96	8.7 (8.4-9.1)	
Karnali province	717	9.4 (9.3-9.6)	
Sudurpashchim Province	724	9.1 (9.0-9.2)	

**BMI(kg/m²)**			
Underweight	372	8.0 (7.8-8.2)	0.000
Normal	2771	8.9 (8.8-8.9)	
Overweight	962	9.7 (9.6-9.8)	
Obese	240	10.6 (10.4-10.9)	
**Hypertension**			
No	3086	9.1 (9.0-9.2)	0.735
Yes	1220	9.1 (9.0-9.2)	
**Diabetes**			
No	3917	9.1 (9.1-9.2)	0.533
Yes	255	9.0 (8.8-9.3)	
**Cholesterol**			
No	3770	9.2 (9.1-9.2)	0.000
Yes	539	8.8 (8.6-8.9)	

Male participants consumed on average 1g more salt than female. Young adult (25-39years) participant ate more salt 0.08g than older (55-69 years) age group participants (P==0.000). Although there was no significant difference observed, more than secondary education participants ate less salt than participants who had primary and secondary education. Salt intake decreases as wealth index of the participant's increases Compared to Province 1, participants living in Bagmati, Gandaki Province ate less salt compared to participants living in

Karnali and Sudurpashchim Province however, there was no significant association was observed among participants residing in Province 2 and Lumbini Province. Salt intake increased by 0.2g as BMI increased (0.19) (P==0.000). Participants who were hypertensive, and had high cholesterol ate less salt than people who had normal blood pressure and cholesterol level (P==0.000). Compared to non-diabetic participants diabetic participants ate less salt although this difference was nonsignificant "Table: 4".

Table 4. Multivariable analysis of Salt Intake with Participants' Characteristics

**Characteristics**	Coefficient (β)	S.E	P-Value	95%-CI	R-Squared	Adjusted R-Squared
Female	Reference					
Male	0.98	0.05	0.000	(0.87- 1.1)		
15-24	Reference					
25-39	0.08	0.08	0.333	(-0.08- 0.23)		
40-54	-0.03	0.09	0.737	(-0.20- 0.14)		
55-69	-0.43	0.10	0.000	(-0.63- -0.24)		
None/less than primary	Reference					
Primary	0.01	0.07	0.846	(-0.12- 0.15)		
Secondary	0.23	0.08	0.756	(-0.12- 0.17)		
More than secondary	-0.11	0.09	0.206	(-0.29- 0.06)		
Lowest	Reference					

Second	0.09	0.07	0.205	(-0.05- 0.24)		
Middle	-0.07	0.08	0.389	(-0.22- 0.09)		
Fourth	-0.19	0.08	0.024	(-0.35- -0.02)		
Highest	-0.31	0.09	0.000	(-0.48- -0.14)	25	24.6
Province 1	Reference					
Province 2	-0.05	0.09	0.543	(-0.22- 0.12)		
Bagmati Province	-0.31	0.09	0.000	(-0.49- -0.14)		
Gandaki Province	-0.20	0.08	0.019	(-0.3- -0.03)		
Lumbini Province	-0.25	0.17	0.153	(-0.59- 0.09)		
Karnali province	0.52	0.09	0.000	(0.34- 0.69)		
Sudurpashchim Province	0.28	0.09	0.001	(0.11- 0.45)		
BMI	0.19	0.01	0.000	(0.18- 0.21)		
Hypertension(No)	Reference					
Hypertension (Yes)	-0.28	0.06	0.000	(-0.40- -0.16)		
Diabetes (No)	Reference					
Diabetes (Yes)	-0.09	0.11	0.405	(-0.29- 0.12)		
High Cholesterol (No)	Reference					
High cholesterol (Yes)	-0.32	0.08	0.000	(-0.47- -0.17)		
Constant	4.53	0.18	0.000	(4.12- 4.87)		

Discussion

To our best knowledge, this is the first study to provide an in-depth evaluation of the capacity of spot urine samples to estimate mean population-level salt intake in Nepalese population using a nationally representative sample. Estimation of salt intake based upon spot urine samples had excellent concordance with 24-hr urine collection measurements [30] and had outstanding sensitivity (97%) and specificity(100%) at classifying mean population salt intake as above or below the World Health Organization maximum guideline value of 5 g/day [31]. Our finding demonstrated the substantial heterogeneity in average population level salt intake in Nepalese population.

We found that mean 24-h salt intake was 9.6g/day for men and 8.7 g/day for women, with mean salt intake 9.1g/day among adult Nepalese population, higher than WHO recommended amount of 5g/day and majority of population (69.4%) in our context consumed more salt than this recommendation. Interestingly, our study reported 31% of the population consumed over 10g/day salt which is double than the WHO recommended value. The average mean salt consumption level in our study is comparable to small scale studies conducted in Nepal [8], in Urban South Indian Population [32], in some states of India [33], in Bhutan [34], and in Korea [35] . The salt intake value obtained in this study is slightly lower than the previous finding reported from Nepal [36], Bangladesh [37], and China [38] which uses 24-hour urine collection measurement method. However; both methods of measurements show salt consumption in Nepal is well above the WHO global recommendation, indicating urgent actions is needed to tackle the non-communicable disease crisis in the country and to take action to reduce population wide dietary salt intake to decrease the number of deaths from hypertension, cardiovascular disease and stroke. In low- and middle-income countries (LMICs) the major contributing factors to the

high amount of daily salt intake come from discretionary salt used during cooking and salting food at the table [39,40]. A study done in south India demonstrated that food items; pulse-based dishes, cereal-based dishes and vegetable-based dishes are the major contributors to daily salt intake [41]. We also observed that similar food habits among us and these all might be the responsible factors for high salt intake in Nepalese population. It has already been reported that intake of low sodium salt is beneficial to health in many aspect [42,43,44]. Thus, it is recommended to create awareness among the general public in LMICs to cut down the use of discretionary salt in foods to decrease the level of salt intake [39,42].

Our finding reported the sodium/potassium ratio for the studied population was 3.4 (SE, 0.2), which is consistent with the finding in previous study done by Samoa et al [45]. Reducing the Na/K ratio is essential for preventing hypertension and cardiovascular disease; however there is no generally accepted recommended guideline for the Na/K ratio [46,47]. Future studies are required to establishing the Na/K ratio for providing information to individuals regarding the risk of hypertension and cardiovascular disease [47]. The finding of this study suggested that higher value of salt intake in male than female which is consistent with other studies [8,32,35,36,45,48,49]. Most of the male population in Nepal is engaged in outdoor activity and most often consume prepared or ready to eat foods which might leads to greater sodium consumption among male than female. This study shows young adult (25-39 years) ate more salt as compared to age group between (15-24 years), however, salt consumption is decline in middle (40-54 years) and older (55-69 years) age group people which is not surprising as similar finding were observed in the study conducted in Nepal [36]. Nepalese economic structure has changed shifting away from agricultural food supply system towards modern processing food

supply system. The trade liberalization has made processed foods easily available at supermarkets and fast food outlets [50]. Our findings also align with the current study conducted in Lifestyle Practices and Obesity in Nepalese Youth which shows majority (75.78%) of respondents consumes fast-food [51]. Similarly, people with higher grade levels were significantly highly likely to be knowledgeable about risk factors of non-communicable disease [52] , which also reflect in this study as salt intake is low in population who had education more than secondary level. Risk factors of non-communicable diseases increased with increasing wealth [53,54], however; in contrast to these findings our study shows salt consumption was decline in those people who were included in fourth and highest wealth index. This may be due to fact that wealthier population may be aware about risk factor of non-communicable disease and may change their lifestyle. Our study showed people who reside in Karnali and Sudhurpashchim province ate more salt than other province. Salt intake is increased as BMI increased in line with another study which shows that salt intake was higher in overweight and obese individuals, this may lead to susceptible to non-communicable disease in later life among those population[33,36,45,55,56]. The correlation of high salt intake with obesity is well known, but the biological mechanisms behind this correlation are not well understood yet. High sodium intake has been suggested as an indirect cause of obesity through increased thirst following consumption of highly salted foods causing an increased intake of sugar-sweetened soft drinks [57,58].

Unlike other studies conducted elsewhere [56,59,60,61], we didn’t find any significant association between increase salt intake and hypertension. It may be due to the fact that hypertensive individuals are aware about the amount of salt intake as they are advised by the treating physician and dietician about the amount of salt intake in their diet. A possible reason

for this is that patient with raised blood pressure and under medication advice to restrict sodium intake or phenotype of salt sensitivity is heterogeneous, influenced from genetic to environmental factors with multiple mechanisms that potentially link high salt intake to increases in blood pressure [62]. However, our finding reported salt intake was less among hypertensive population compared to non-hypertensive. Sodium reduction substantially lowered blood pressure, even among those with starting systolic blood pressure levels as low as 120 mm Hg [63]. These findings indicate potentially important health benefits from sodium reduction among normotensive as well as hypertensive individuals [63]. More importantly, sodium reduction among normotensive individuals could potentially avert or delay the development of hypertension with ageing, as the association between sodium intake and blood pressure is greater at older age [64]. High-salt intake is a major risk factor for developing hypertension in type 2 diabetes mellitus, but its effects on glucose homeostasis are controversial [65] . Similarly, we did not find the association of salt intake in diabetes population though there was decrease in salt intake in population who are diabetic than non-diabetic. This study finds association between salt intake and blood lipids, however there is no established conclusive evidence regarding the relationship between sodium intake and blood lipid level [61,66,67,68]. Study has shown mixed evidence on reducing sodium intake may had or no have significant adverse effect on blood lipids and association between sodium intake and all-cause mortality, incident cardiovascular disease and non-fatal coronary heart disease [61,69,70]. Our study clearly highlighted the need of further studies using longitudinal data or randomized clinical trials on role of dietary salt in the development / prevention of blood pressure, diabetes mellitus and cholesterol levels in Nepalese population taking considerations of different measures of population characteristics.

This study has several strength, first of it analyzed first time the salt intake in community level in Nepalese population, and is not limited to any particular region, caste, sex, and age. Second, to our best knowledge, this is the first study in Nepalese population which demonstrated the association of salt intake with different characteristic however, this study has also few major limitation; being a cross sectional study, it cannot attribute the causality from the association of estimated salt intake and BMI and blood pressure. Second, the use of spot urine sample for the estimation of salt intake instead of its gold standard method 24-h urine sample. Therefore, its validation study in small Nepalese population is recommended. However, many large epidemiological studies have already adopted the estimation of salt intake using spot urine sample instead of 24-h urine sample because of the ease of urine sample collection and participant enrollment. Third, this study has included hypertensive individual but their detailed information on antihypertensive drug was not inclusively collected via a questionnaire. It is postulated that some antihypertensive drugs with natriuretic properties could be a confounder, but the other antihypertensive drug could not be the confounder. To be sure about the confounding factor, a correlation study with exposure namely salt intake in this study is required. Forth, we have not focused on status of renal function tests (RFT) of the participants which might have influence on estimation of salt intake.

WHO is now considering using spot urine samples to assess population salt intake as part of STEPS in order to monitor progress toward the global targets. Several equations have been developed and tested and proven potentially useful for estimating mean population 24-hour salt intake from spot urine samples [21,24,25,27]. However, they have yet to be tested and evaluated in Nepalese population. The estimation of salt intake using spot urine samples is likely to

discriminate that salt intakes are well above the WHO recommendations and may provide a as a benchmark to assess the impact of salt reduction efforts in Nepal [19].

Conclusions

Our study finding suggested that higher estimated daily salt intake, approximately double than the limit recommended by WHO. A total of 69.4% of the population had a salt intake >5 g/d. This study demonstrated the association of high salt intake with different health indicators and documented the importance of lifestyle modification on health indicators. These findings suggest a need of urgent actions to reduce salt consumption in Nepal to achieve the global target of a 30% reduction in population salt intake by 2025. Population-based salt reduction strategies are cost-effective and cost-saving in most of the settings for prevention of non-communicable diseases.

Acknowledgements

We would like to acknowledge the effort of all the individuals involved in this survey, express my deep sense of appreciation to the steering committee and technical working group (TWG) member. We would like to express my sincere thanks to Dr. Manju Rani, Regional Advisor (Non-communicable diseases policy, governance and surveillance), Naveen Agarwal (Surveillance Management Associate at WHO/SEARO); Dr. Patricia Rarau (WHO HQ) (training of field enumerators); Dr. Stefan Savin (WHO HQ) (data analysis); Dr. Md. Khurshid Alam Hyder (WHO Nepal) and Dr. Lonim Prasai Dixit (WHO Nepal), Ms. Yvonne Y. Xu, Ms.

Preetika D. Banerjee, Ms. Surabhi Chaturvedi, from WHO SEARO for their valuable and remarkable contribution from beginning to the end of this survey. Lastly, we would like to thanks all NHRC staff that helped during conducting this study.

References

1. Morris MJ, Na ES, Johnson AK (2008) Salt craving: the psychobiology of pathogenic sodium intake. Physiology & behavior 94: 709-721.
2. Gupta N, JANI KK, Gupta N (2011) Hypertension: salt restriction, sodium homeostasis, and other ions. Indian journal of medical sciences 65.
3. Kaushik S, Kumar R, Kain P (2018) Salt an Essential Nutrient: Advances in Understanding Salt Taste Detection Using Drosophila as a Model System. Journal of experimental neuroscience 12: 1179069518806894.
4. Cappuccio FP (2011) Cardiovascular and other effects of salt consumption. Kidney Int Suppl 3: 312-315.
5. Organization WH (2010) Regional Consultation on Strategies to Reduce Salt Intake, Singapore, 2-3 June 2010: report. Manila: WHO Regional Office for the Western Pacific.
6. Kontis V, Mathers CD, Rehm J, Stevens GA, Shield KD, et al. (2014) Contribution of six risk factors to achieving the 25× 25 non-communicable disease mortality reduction target: a modelling study. The Lancet 384: 427-437.
7. Ha SK (2014) Dietary salt intake and hypertension. Electrolytes & Blood Pressure 12: 7-18.
8. Ghimire K, Adhikari TB, Rijal A, Kallestrup P, Henry ME, et al. (2019) Knowledge, attitudes, and practices related to salt consumption in Nepal: Findings from the community-based management of non-communicable diseases project in Nepal (COBIN). The Journal of Clinical Hypertension 21: 739-748.
9. Riley L, Cowan M (2014) Noncommunicable diseases country profiles 2014. Geneva: World Health Organization.
10. Dhimal M BB, Bhattarai S, Dixit LP, Hyder MKA, Agrawal N, Rani M, Jha AK (2020) Report of Non Communicable Disease Risk Factors STEPS Survey Nepal 2019. . Kathmandu: Nepal Health Research Council.
11. Dhungana RR, Devkota S, Khanal MK, Gurung Y, Giri RK, et al. (2014) Prevalence of cardiovascular health risk behaviors in a remote rural community of Sindhuli district, Nepal. BMC cardiovascular disorders 14: 92.

12. Aryal A, Citrin D, Halliday S, Kumar A, Nepal P, et al. (2020) Estimated cost for cardiovascular disease risk-based management at a primary healthcare center in Nepal. Global health research and policy 5: 2.
13. Organization WH (2012) A comprehensive global monitoring framework including indicators and a set of voluntary global targets for the prevention and control of noncommunicable diseases. Geneva: World Health Organization.
14. Sharma SR, Page R, Matheson A, Lambrick D, Faulkner J, et al. (2019) Non-communicable disease prevention in Nepal: systemic challenges and future directions. Global health promotion 26: 94-97.
15. Organization WH (2007) Reducing salt intake in populations: report of a WHO forum and technical meeting, 5-7 October 2006, Paris, France.
16. McLean RM (2014) Measuring population sodium intake: a review of methods. Nutrients 6: 4651-4662.
17. Organization WH (2011) Strategies to monitor and evaluate population sodium consumption and sources of sodium in the diet: report of a joint technical meeting convened by WHO and the Government of Canada.
18. Simpson F (1988) Sodium intake, body sodium, and sodium excretion. The Lancet 332: 25-29.
19. Huang L, Crino M, Wu JH, Woodward M, Barzi F, et al. (2016) Mean population salt intake estimated from 24-h urine samples and spot urine samples: a systematic review and meta-analysis. International journal of epidemiology 45: 239-250.
20. Wilson T, Garcia-Perez I, Posma JM, Lloyd AJ, Chambers ES, et al. (2019) Spot and cumulative urine samples are suitable replacements for 24-hour urine collections for objective measures of dietary exposure in adults using metabolite biomarkers. The Journal of nutrition 149: 1692-1700.
21. Brown IJ, Dyer AR, Chan Q, Cogswell ME, Ueshima H, et al. (2013) Estimating 24-hour urinary sodium excretion from casual urinary sodium concentrations in Western populations: the INTERSALT study. American journal of epidemiology 177: 1180-1192.
22. Rhee M-Y, Kim J-H, Shin S-J, Gu N, Nah D-Y, et al. (2014) Estimation of 24-hour urinary sodium excretion using spot urine samples. Nutrients 6: 2360-2375.
23. Ji C, Miller M, Venezia A, Strazzullo P, Cappuccio F (2014) Comparisons of spot vs 24-h urine samples for estimating population salt intake: validation study in two independent samples of adults in Britain and Italy. Nutrition, Metabolism and Cardiovascular Diseases 24: 140-147.
24. Toft U, Cerqueira C, Andreasen AH, Thuesen BH, Laurberg P, et al. (2014) Estimating salt intake in a Caucasian population: can spot urine substitute 24-hour urine samples? European journal of preventive cardiology 21: 1300-1307.
25. Tanaka T, Okamura T, Miura K, Kadowaki T, Ueshima H, et al. (2002) A simple method to estimate populational 24-h urinary sodium and potassium excretion using a casual urine specimen. Journal of human hypertension 16: 97-103.
26. Mann SJ, Gerber LM (2010) Estimation of 24-hour sodium excretion from spot urine samples. The Journal of Clinical Hypertension 12: 174-180.

27. Kawasaki T, Itoh K, Uezono K, Sasaki H (1993) A simple method for estimating 24 h urinary sodium and potassium excretion from second morning voiding urine specimen in adults. Clinical and experimental pharmacology and physiology 20: 7-14.
28. National Academies of Sciences E, Medicine (2019) Dietary Reference Intakes for sodium and potassium: National Academies Press.
29. Organization WH (2013) Expert meeting on population sodium reduction strategies for prevention and control of noncommunicable diseases in the South-East Asia Region, 11-13 December 2012. World Health Organization. Regional Office for South-East Asia.
30. Mizéhoun-Adissoda C, Houehanou C, Chianéa T, Dalmay F, Bigot A, et al. (2016) Estimation of Daily Sodium and Potassium Excretion Using Spot Urine and 24-Hour Urine Samples in a Black Population (Benin). J Clin Hypertens 18: 634-640.
31. Huang L, Crino M, Wu JH, Woodward M, Barzi F, et al. (2016) Mean population salt intake estimated from 24-h urine samples and spot urine samples: a systematic review and meta-analysis. Int J Epidemiol 45: 239-250.
32. Radhika G, Sathya R, Sudha V, Ganesan A, Mohan V (2007) Dietary salt intake and hypertension in an urban south Indian population–[CURES-53]. Journal of Association of Physicians of India 55: 405-411.
33. Johnson C, Mohan S, Rogers K, Shivashankar R, Thout SR, et al. (2017) Mean dietary salt intake in urban and rural areas in India: a population survey of 1395 persons. Journal of the American Heart Association 6: e004547.
34. Organization WH (2016) National survey for noncommunicable disease risk factors and mental health using WHO STEPS approach in Bhutan-2014.
35. Kim YC, Koo HS, Kim S, Chin HJ (2014) Estimation of daily salt intake through a 24-hour urine collection in Pohang, Korea. J Korean Med Sci 29: S87-S90.
36. Neupane D, Rijal A, Henry ME, Kallestrup P, Koirala B, et al. (2020) Mean dietary salt intake in Nepal: A population survey with 24-hour urine collections. The Journal of Clinical Hypertension 22: 273-279.
37. Zaman MM, Choudhury SR, Ahmed J, Khandaker RK, Rouf MA, et al. (2017) Salt intake in an adult population of Bangladesh. Global heart 12: 265.
38. Tan M, He FJ, Wang C, MacGregor GA (2019) Twenty-four-hour urinary sodium and potassium excretion in China: a systematic review and meta-analysis. Journal of the American Heart Association 8: e012923.
39. Menyanu E, Russell J, Charlton K (2019) Dietary Sources of Salt in Low- and Middle-Income Countries: A Systematic Literature Review. International journal of environmental research and public health 16: 2082.
40. Fathima KA, Bhargava M (2018) Salt reduction and low-sodium salt substitutes: Awareness among health-care providers in Mangalore, Karnataka. Indian journal of community medicine: official publication of Indian Association of Preventive & Social Medicine 43: 266.
41. Ravi S, Bermudez OI, Harivanzan V, Kenneth Chui KH, Vasudevan P, et al. (2016) Sodium Intake, Blood Pressure, and Dietary Sources of Sodium in an Adult South Indian Population. Ann Glob Health 82: 234-242.

42. He FJ, MacGregor GA (2009) A comprehensive review on salt and health and current experience of worldwide salt reduction programmes. Journal of human hypertension 23: 363-384.
43. He FJ, MacGregor GA (2010) Reducing Population Salt Intake Worldwide: From Evidence to Implementation. Progress in Cardiovascular Diseases 52: 363-382.
44. Cobb LK, Appel LJ, Anderson CAM (2012) Strategies to reduce dietary sodium intake. Current treatment options in cardiovascular medicine 14: 425-434.
45. Webster J, Su'a SAF, Ieremia M, Bompoint S, Johnson C, et al. (2016) Salt intakes, knowledge, and behavior in samoa: monitoring salt-consumption patterns through the World Health Organization's surveillance of noncommunicable disease risk factors (STEPS). The Journal of Clinical Hypertension 18: 884-891.
46. Iwahori T, Miura K, Ueshima H (2017) Time to consider use of the sodium-to-potassium ratio for practical sodium reduction and potassium increase. Nutrients 9: 700.
47. Iwahori T, Miura K, Ueshima H, Tanaka-Mizuno S, Chan Q, et al. (2019) Urinary sodium-to-potassium ratio and intake of sodium and potassium among men and women from multiethnic general populations: the INTERSALT Study. Hypertension Research 42: 1590-1598.
48. Xu J, Wang M, Chen Y, Zhen B, Li J, et al. (2014) Estimation of salt intake by 24-hour urinary sodium excretion: a cross-sectional study in Yantai, China. BMC public health 14: 1-6.
49. Jensen PN, Bao TQ, Huong TTT, Heckbert SR, Fitzpatrick AL, et al. (2018) The association of estimated salt intake with blood pressure in a Viet Nam national survey. PloS one 13: e0191437.
50. Subedi YP, Marais D, Newlands D (2017) Where is Nepal in the nutrition transition? Asia Pacific journal of clinical nutrition 26: 358.
51. Nepal G, Tuladhar ET, Dahal S, Ahamad ST, Adhikari S, et al. (2018) Lifestyle practices and obesity in Nepalese youth: a cross-sectional study. Cureus 10.
52. Onagbiye SO, Tsolekile LP, Puoane T (2020) Knowledge of non-communicable disease risk factors among community health workers in South Africa. The Open Public Health Journal 13.
53. Schneider M, Bradshaw D, Steyn K, Norman R, Laubscher R (2009) Poverty and non-communicable diseases in South Africa. Scandinavian journal of public health 37: 176-186.
54. Biswas T, Islam MS, Linton N, Rawal LB (2016) Socio-economic inequality of chronic non-communicable diseases in Bangladesh. PloS one 11: e0167140.
55. Ma Y, He FJ, MacGregor GA (2015) High salt intake: independent risk factor for obesity? Hypertension 66: 843-849.
56. Thuesen BH, Toft U, Buhelt LP, Linneberg A, Friedrich N, et al. (2015) Estimated daily salt intake in relation to blood pressure and blood lipids: the role of obesity. European journal of preventive cardiology 22: 1567-1574.
57. Grimes CA, Riddell LJ, Campbell KJ, Nowson CA (2013) Dietary salt intake, sugar-sweetened beverage consumption, and obesity risk. Pediatrics 131: 14-21.
58. Karppanen H, Mervaala E (2006) Sodium intake and hypertension. Progress in cardiovascular diseases 49: 59-75.

59. Mente A, O'Donnell MJ, Rangarajan S, McQueen MJ, Poirier P, et al. (2014) Association of urinary sodium and potassium excretion with blood pressure. New england journal of medicine 371: 601-611.
60. He FJ, Li J, MacGregor GA (2013) Effect of longer term modest salt reduction on blood pressure: Cochrane systematic review and meta-analysis of randomised trials. bmj 346.
61. Graudal NA, Hubeck-Graudal T, Jurgens G (2020) Effects of low sodium diet versus high sodium diet on blood pressure, renin, aldosterone, catecholamines, cholesterol, and triglyceride. Cochrane Database of Systematic Reviews.
62. Choi HY, Park HC, Ha SK (2015) Salt Sensitivity and Hypertension: A Paradigm Shift from Kidney Malfunction to Vascular Endothelial Dysfunction. Electrolyte & blood pressure : E & BP 13: 7-16.
63. Huang L, Trieu K, Yoshimura S, Neal B, Woodward M, et al. (2020) Effect of dose and duration of reduction in dietary sodium on blood pressure levels: systematic review and meta-analysis of randomised trials. bmj 368.
64. Group ICR (1988) Intersalt: an international study of electrolyte excretion and blood pressure. Results for 24 hour urinary sodium and potassium excretion. BMJ: British Medical Journal: 319-328.
65. Takagi Y, Sugimoto T, Kobayashi M, Shirai M, Asai F (2018) High-salt intake ameliorates hyperglycemia and insulin resistance in WBN/Kob-Leprfa/fa rats: a new model of type 2 diabetes mellitus. Journal of diabetes research 2018.
66. Organization WH (2012) Guideline: Sodium intake for adults and children: World Health Organization.
67. Graudal NA, Galløe AM, Garred P (1998) Effects of sodium restriction on blood pressure, renin, aldosterone, catecholamines, cholesterols, and triglyceride: a meta-analysis. Jama 279: 1383-1391.
68. Graudal NA, Hubeck-Graudal T, Jurgens G (2011) Effects of low sodium diet versus high sodium diet on blood pressure, renin, aldosterone, catecholamines, cholesterol, and triglyceride. Cochrane Database Syst Rev 9.
69. Hooper L, Bartlett C, Davey SG, Ebrahim S (2004) Advice to reduce dietary salt for prevention of cardiovascular disease. Cochrane Database Syst Rev 1.
70. Strazzullo P, D'Elia L, Kandala NB, Cappuccio FP (2009) Salt intake, stroke, and cardiovascular disease: meta-analysis of prospective studies. bmj 24.
