# Supplementary material for: Estimation of mean population salt intakes using spot urine samples and associations with body mass index, hypertension, raised blood sugar and hypercholesterolemia: Findings from STEPS Survey 2019, Nepal
Source: PLoS One. 2022 Apr 12;17(4):e0266662. doi: 10.1371/journal.pone.0266662 (PMC9004746; doi:10.1371/journal.pone.0266662)
Supplement: Attachment — Submitted filename: PONE-D-21-06676_reviewer.docx [file pone.0266662.s001.docx]

This study aims to determine the population mean of salt intakes and its correlates using 2019 STEPS survey data in Nepal. The research question is adequately motivated. However, there are some concerns that authors need to address to improve the quality of the manuscript.

Comments

- The title and objectives of the study are not consistent.
- Title needs some improvement. For example, while hypertension and raised blood sugar indicate a direction, the authors did not mention about BMI and lipid level similarly. Please revise the title. Also, use hypercholesterolemia instead of high cholesterol level.
- It is highly preferable to use consistent terms throughout the whole manuscript. For instance, raised blood sugar is used in the title but there is no indication of such variables in the result, except diabetes.
- The authors mentioned that the equation from INTERSALT was developed using a large heterogenous population sample. Do the authors mean by the term “heterogenous” that the equation can be applied to the Nepalese population?
- The authors also explicitly mentioned the equation INTERSALT for southern Europe. If this is the case, there would be at least some anthropometric differences between these and Nepalese population? For example, considering BMI and age adjustment to the equation would seem to be more appropriate instead of using the original equation.
- In Table 2, the prevalence of salt intake > 5 g was higher in women than in man, whereas that of salt intake > 10 g was reversed, higher in man than in women. Please discuss this.
- As the authors rightly discussed that people with history of hypertension may be aware of their hypertensive status and likely to reduce salt intake. This can also be linked to behavioral and lifestyle choices. That’s why salt intake is higher in men as women are more likely to take care of themselves. Likewise, BMI can also be attributed by lifestyle choices and hence, by salt intake. Therefore, there is no clear indication of exposure and outcome pathway in this model.
- What are the clinical implications of the study beyond the assessment of population salt intake and validation of equation in Nepalese population?
- Conclusion should be based on the study findings. For instance, the study’s results did not present the lifestyle modification and its importance on health indicators. It is also not a finding of the study that “population-based salt reduction strategies are cost-effective and cost-saving in most of the settings for prevention of non-communicable diseases.”. Please revise the conclusion.
- References should be relevant and up-to-date. Please try to reduce the number of references.
- in Table 4, there is no need to report R squared or adjusted R squared without presenting all models. Remove the last two columns.
- If p-value is 0.000, indicate using “<0.001” in Table 3 and 4.
