# Supplementary material for: Estimation of mean population salt intakes using spot urine samples and associations with body mass index, hypertension, raised blood sugar and hypercholesterolemia: Findings from STEPS Survey 2019, Nepal
Source: PLoS One. 2022 Apr 12;17(4):e0266662. doi: 10.1371/journal.pone.0266662 (PMC9004746; doi:10.1371/journal.pone.0266662)
Supplement: Attachment — Submitted filename: Response to reviewes.docx [file pone.0266662.s004.docx]

24 August 2021

Shyam Sundar Budhathoki, MBBS, MD, MPH
Academic Editor
PLOS ONE

PONE-D-21-06676R1
Estimation of mean Population salt intakes using Spot Urine samples and association with Body Mass Index, Hypertension, Raised Blood Sugar and Blood Lipids: Findings from the Non-communicable Disease Risk Factors (STEPS) Survey 2019 in Nepal

Dear Dr. Budhathoki

Thank you very much for your email of 03 August 2021 and comments on our manuscript. We have carefully revised the manuscript in response to the extensive and insightful comments we received from the reviewer.

We noticed that the reviewer had commented on our originally submitted version and we have already addressed many comments on the revised manuscript (R1). In particular, the reviewer provided constructive comments with some corrections. In the revised manuscript version, all the suggested corrections are made. We have also revised the content of introduction, methodology, result and discussion section according to reviewers’ advice and have paid special attention to correcting all typological errors. Our revised manuscript is also edited by native speaker from UK. Appended below is the list of all yours and reviewer comments along with our responses to each point.

We hope that this revised version will be suitable for publication in PLoS ONE.

Yours sincerely,

Meghnath Dhimal

**Review Comments to the Author**

**1. May be running title reflect the main aim of the paper i.e. estimating mean population salt intake and associated factors...**

** Thank you for your suggestion and instructions; the running title has been revised to “**Estimating mean population salt intake and associated factors**”. (Lines: 7)

**2. Accurate measurement or population data or both??**

**Thank you for your suggestion. We have revised the text accordingly. (Lines: 35).

**3. What does for the first time mean? Is it "in STEPS survey or NHRC research or first time in Nepal??" May be remove it from abstract. Explain in main text.**

**Thank you for your suggestion. Mean salt estimation was carried out in Nepal for the first time. We have remove the first time in abstract and mentioned it in main text.

**4. I think the main purpose of this study was to estimate population level data to support public health strategies to reduce salt intake; not to establish a causal association of salt intake, right?**

**Thank you for your constructive feedback and suggestion. We have removed the statement and revised the statement as follows. (Line: 59-61).

“Furthermore, these findings build a strong case of actions needed to reduce salt consumption in Nepal to achieve the global target of a 30% reduction in population salt intake by 2025.”

**5. Please simplify the Sentence as it is unclear may be in 2 sentences.**

**Thank you for your suggestion. We have revised the text accordingly. (Lines: 74).

**6. How does the disease put economic burden? Please add one or two sentence.**

**Thank you for your suggestion. We have added the following text in the manuscript. (Lines: 88-90).

“NCDs incurred economic burden by increasing health-care costs, lowering workplace productivity, increasing sick days, and inflicting permanent disability”.

**7. I think this name of not complete action plan for what? Control of NCDs.**

**Thank you for your constructive feedback. The sentence is revised in main text (Lines: 94-95).

**8.** **I think the authors should focus on utility and practicality of spot collection/test…Challenges of 24h urine collection/test such as “may miss the sodium estimation excreted through non-urinary routes and also applies to spot collection/test; maybe remove irrelevant sentences.**

**Thank you for your feedback and suggestions. We had addressed those comments on revised manuscript (Line: 99-112).

“The standard approach to measuring the mean salt intake of a population has been the collection of 24-h urine samples on a subset of individuals.  However, this method is troublesome, time-consuming, costly to participants due to the complex nature of urine sample collection and may miss the sodium estimation excreted through non-urinary routes and some dietary sodium derives from sources other than salt. Equations that use spot urine samples to estimate population salt intake have been explored as a possible alternative in a number of studies. WHO included spot urine in STEPs survey protocol in December 2013, as a measure to estimate mean population salt intake”.

**9. Why are these references not written as 21-28??**

**Thank you for your suggestion. We have corrected the references. (Line: 111)

**10. Methods**

** Thank you for your valuable and constructive suggestion and instructions in methodological section; we had addressed those comments on revised manuscript submitted after first revision, however; we received the comments on manuscript that was submitted at beginning. However; any missing revisions that were raised has been revised accordingly in methodological section.

**11.** **Was there any active attempt to identify how many were under hypertension medication or have received counseling from health workers and how many were taking their BP measurements for the first time?**

**Thank you for your query. We have collected all the information as mentioned above which can be found in Survey report. Link: <https://www.who.int/ncds/surveillance/steps/2012-13_Nepal_STEPS_Report.pdf>.

**12. I think just discussing significant association is enough; no need to stress about lack of association of salt intake with metabolic factors. The key aspect of this research is mean salt intake and how this could inform public health measures in Nepal.**

** Thank you for your suggestions and helpful criticism. We have handled such issues on amended manuscripts submitted after first revision, as previously stated; however, we had received comments on manuscripts submitted during the first round. Any missing revisions that were raised, however, have been addressed in both the discussion and conclusion sections. In conclusion section, we have revised the statement as follows. (Line: 453-459).

“According to our findings, Nepal has a daily salt intake that is around double that of the WHO's recommended limit. A total of 70.8% of the population had a salt intake >5 g/d. The findings of this study will aid policymakers and other important stakeholders in Nepal at the central, federal, and local levels in tracking trends and progress toward salt intake targets, as well as guiding future policies and initiatives aimed at reducing salt intake among the population. Furthermore, these findings build a strong case of actions needed to reduce salt consumption in Nepal to achieve the global target of a 30% reduction in population salt intake by 2025”.
